# Mendelian randomization study supports the causal effects of air pollution on longevity via multiple age-related diseases

**DOI:** 10.1038/s41514-023-00126-0

**Published:** 2023-12-19

**Authors:** Shizheng Qiu, Yang Hu, Guiyou Liu

**Affiliations:** 1 School of Computer Science and Technology, Harbin Institute of Technology, Harbin, 150001 China; 2 Beijing Institute of Brain Disorders, Laboratory of Brain Disorders, Ministry of Science and Technology, Collaborative Innovation Center for Brain Disorders, Capital Medical University, Beijing, 100069 China; 3 Chinese Institute for Brain Research, Beijing, China; 4 Key Laboratory of Cerebral Microcirculation in Universities of Shandong; Department of Neurology, Second Affiliated Hospital; Shandong First Medical University & Shandong Academy of Medical Sciences, Taian, 271000 Shandong China; 5 Beijing Key Laboratory of Hypoxia Translational Medicine, National Engineering Laboratory of Internet Medical Diagnosis and Treatment Technology, Xuanwu Hospital, Capital Medical University, Beijing, 100053 China

**Keywords:** Risk factors, Epidemiology, Ageing

## Abstract

Growing evidence suggests that exposure to fine particulate matter (PM_2.5_) may reduce life expectancy; however, the causal pathways of PM_2.5_ exposure affecting life expectancy remain unknown. Here, we assess the causal effects of genetically predicted PM_2.5_ concentration on common chronic diseases and longevity using a Mendelian randomization (MR) statistical framework based on large-scale genome-wide association studies (GWAS) (>400,000 participants). After adjusting for other types of air pollution and smoking, we find significant causal relationships between PM_2.5_ concentration and angina pectoris, hypercholesterolaemia and hypothyroidism, but no causal relationship with longevity. Mediation analysis shows that although the association between PM_2.5_ concentration and longevity is not significant, PM_2.5_ exposure indirectly affects longevity via diastolic blood pressure (DBP), hypertension, angina pectoris, hypercholesterolaemia and Alzheimer’s disease, with a mediated proportion of 31.5, 70.9, 2.5, 100, and 24.7%, respectively. Our findings indicate that public health policies to control air pollution may help improve life expectancy.

## Introduction

Most of the world’s population is affected by air pollution^1^. Air pollutants, especially fine particulate matter pollution (PM_2.5_), pose a major threat to human health^2^. Previous studies have confirmed that long-term exposure to PM_2.5_ increases the risk of a wide range of chronic diseases and may cause premature death in parts of the population^2–7^. A noteworthy large cross-sectional study conducted in the United States revealed that a reduction of 1 μg/m^3^ in PM_2.5_ exposure corresponded to an increase in life expectancy of 0.12 years^8^. Another population-based cohort of 2.7 million adults in Canada supported the potential public health benefits of air quality interventions^4^. However, the plethora of confounding factors affecting life expectancy makes it difficult to make causal inferences^9^. For instance, socioeconomic factors, such as socioeconomic status (SES), educational attainment, income, and occupation, play a significant role as confounding variables in longevity studies^10–12^. SES can have a substantial impact on various aspects of individuals’ lives, including access to healthcare, living conditions, lifestyle choices, and exposure to environmental hazards, all of which can influence longevity outcomes^13^. Other environmental factors, such as noise pollution and access to green spaces may influence longevity outcomes independently of the exposure being studied^13^. In addition, the mediating pathway by which PM_2.5_ exposure affects longevity remains unknown.

Growing evidence suggests that air pollution affects individuals to varying degrees, and genetic polymorphisms play a significant role in this phenomenon^14,15^. Certain genetic variants may increase susceptibility to the detrimental effects of air pollutants, while others may offer some level of protection. It has been discovered that alleles in the human genome, which mitigate smoke damage, have been present for at least 550,000 years^16^. The impact of air pollution on longevity can be influenced by gene polymorphism in candidate genes associated with longevity, such as *SIRT1* and *FOXO3*^15,17^. Additionally, genetic variations can affect an individual’s inflammatory and oxidative stress responses, which are critical mechanisms in the body’s reaction to air pollution and, therefore, can influence the risk of adverse health outcomes and potentially reduce longevity^18,19^. Moreover, the heritability of longevity is considerably high, with genetic factors accounting for approximately one-third of the variability in human lifespan^20^. Consequently, genetic data can be employed to investigate the causal relationship between PM_2.5_ concentration and longevity outcomes.

**Fig. 1:**
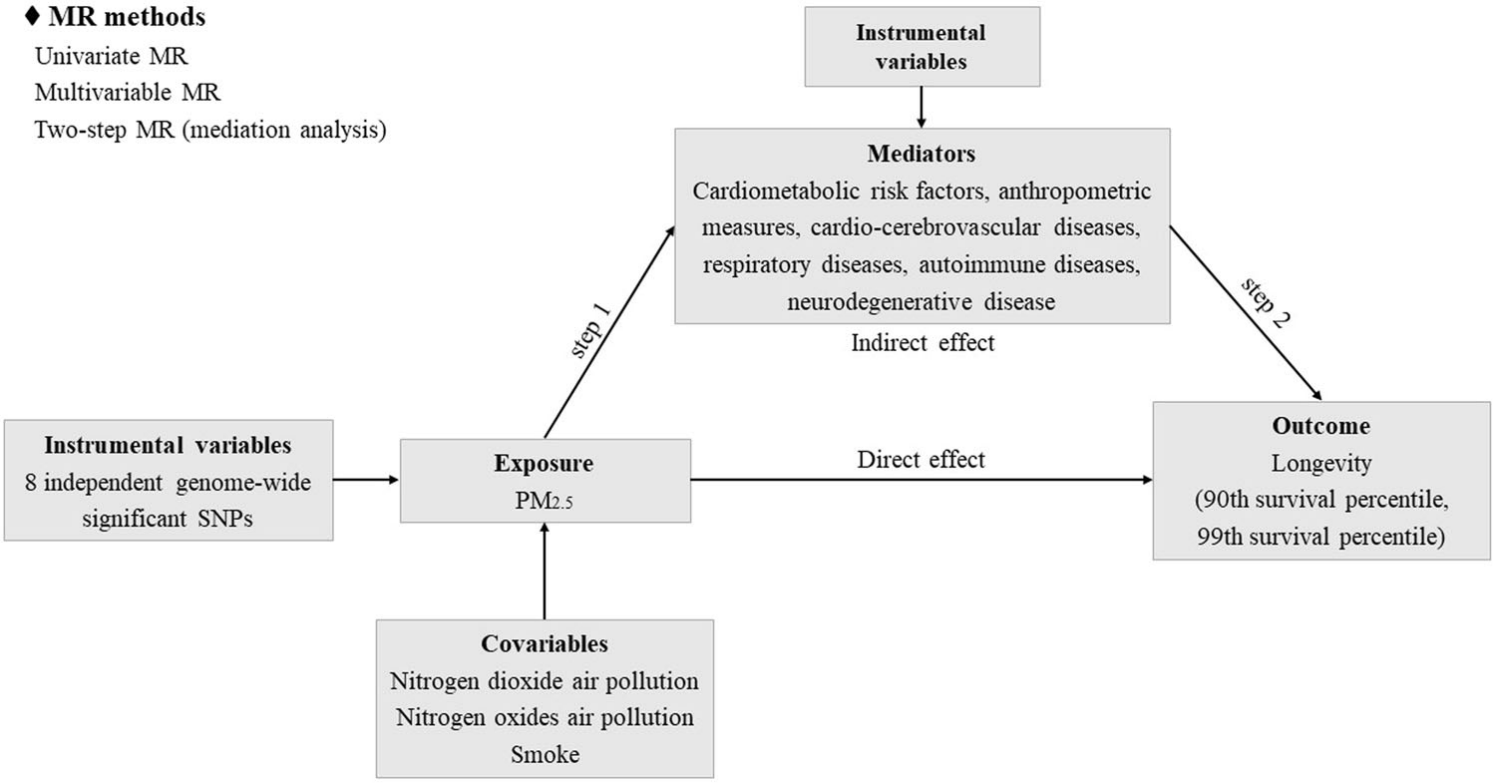
Study design.

In the absence of randomized controlled trials (RCTs), we designed a Mendelian randomization (MR) statistical framework using large-scale genome-wide association studies (GWAS). MR utilized genetic variants as instrumental variables to assess the causal relationship between exposure and outcome^21–26^. The randomly allocated process of alleles of genetic variants was used to simulate RCTs^27,28^. The alleles of these instruments were determined at the time of meiosis and fertilization, thereby minimizing issues of confounding factors and reverse causality^27^. Here, we used univariate MR to assess the causal relationship between genetically predicted PM_2.5_ concentration and longevity. We utilized multivariate MR to adjust for the effects of other types of air pollution and smoking on causal estimates. Finally, we conducted a two-step MR analysis (mediation analysis) to explore whether PM_2.5_ concentration influenced longevity via mediating factors (cardiometabolic risk factors, cardio-cerebrovascular diseases, respiratory diseases, autoimmune diseases, and neurodegenerative disease). The conceptual framework was depicted in Fig. 1.

## Results

### Univariable MR

**Fig. 2:**
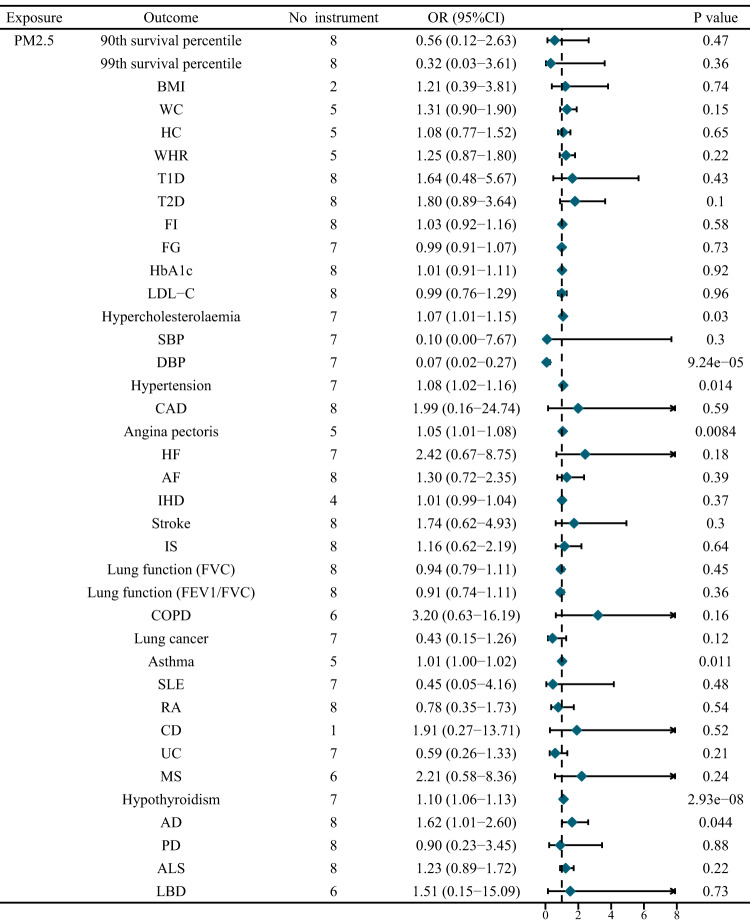
Univariate MR results for the causal relationship of PM_2.5_ concentration on potential mediators and longevity. AD Alzheimer’s disease, AF atrial fibrillation, ALS amyotrophic lateral sclerosis, BMI body mass index, CAD coronary artery disease, CD Crohn’s disease, COPD chronic obstructive pulmonary disease, DBP diastolic blood pressure, FEV1/FVC 1 s forced expiratory volume/FVC, FG fasting glucose, FI fasting insulin, FVC forced vital capacity, HbA1c glycated hemoglobin, HC hip circumference, HF heart failure, IHD ischemic heart disease, IS ischemic stroke, LBD lewy body dementia, MS multiple sclerosis, PD Parkinson’s disease, RA rheumatoid arthritis, SBP systolic blood pressure, SLE Systemic lupus erythematosus, T1D type 1 diabetes, T2D type 2 diabetes, UC Ulcerative colitis, WC waist circumference, WHR: waist-hip ratio.

We performed a univariate MR analysis to investigate the total causal effect of genetically predicted PM_2.5_ concentration on longevity. A total of eight independent genome-wide significant genetic variants were used as instrumental variables, with no weak instruments (F statistic < 10) (Supplementary Table 1). Univariable MR analysis showed a non-significant causal relationship between genetically predicted PM_2.5_ concentration and longevity [90th percentile: Odds Ratio (OR) = 0.56, 95% CI = 0.12 to 2.63, *P* = 0.47; 99th percentile: OR = 0.32, 95% CI = 0.03 to 3.61, *P* = 0.36] (Fig. 2, Supplementary Table 2, Supplementary Figure 1). Genetically predicted PM_2.5_ concentration was also not associated with longevity in sensitivity analysis.

Although PM_2.5_ concentration did not directly affect human life span, it significantly affected several potential mediators and might further indirectly affect human life span. As a result, genetically predicted PM_2.5_ concentration was causally associated with diastolic blood pressure (DBP), hypercholesterolaemia, hypertension, angina pectoris, asthma, hypothyroidism, and Alzheimer’s disease (AD) (Fig. 2, Supplementary Table 3). Genetically predicted PM_2.5_ concentration had no causal effect estimate consistent with body mass index (BMI), hip circumference (HC), waist circumference (WC), waist-hip ratio (WHR), type 1 diabetes (T1D), type 2 diabetes (T2D), fasting glucose (FG), fasting insulin (FI), glycated hemoglobin (HbA1c), low density lipoprotein cholesterolsystolic (LDL-C), blood pressure (SBP), coronary artery disease (CAD), heart failure (HF), atrial fibrillation (AF), ischemic heart disease (IHD), stroke, ischemic stroke (IS), forced vital capacity (FVC), 1 s forced expiratory volume (FEV1)/FVC, chronic obstructive pulmonary disease (COPD), lung cancer, systemic lupus erythematosus (SLE), ulcerative colitis (UC), Crohn’s disease (CD), rheumatoid arthritis (RA), multiple sclerosis (MS), amyotrophic lateral sclerosis (ALS), Parkinson’s disease (PD) and lewy body dementia (LBD). Specifically, for each unit (1.06 micro-g/m^3^) increase in PM_2.5_ exposure, the risk of hypercholesterolaemia increased by 7% (OR = 1.07, 95% CI = 1.01–1.15, *P* = 0.03), the risk of hypertension increased by 8% (OR = 1.08, 95% CI = 1.02–1.16, *P* = 0.014), the risk of angina pectoris increased by 5% (OR = 1.05, 95% CI = 1.01–1.08, *P* = 0.0084), the risk of asthma increased by 1% (OR = 1.01, 95% CI = 1.00–1.02, *P* = 0.011), the risk of hypothyroidism increased by 10% (OR = 1.10, 95% CI = 1.06–1.13, *P* = 2.93E − 08), and the risk of AD increased by 10% (OR = 1.62, 95% CI = 1.01 to 2.60, *P* = 0.044) (Fig. 2, Supplementary Table 3). Genetically predicted PM_2.5_ concentration was significantly associated with reduced DBP (OR = 0.07, 95% CI = 0.02–0.27, *P* = 9.24E − 05).

The sensitivity tests essentially replicated the results of inverse variance weighted (IVW) analysis (Supplementary Table 3). Notably, genetically predicted PM_2.5_ concentration was associated with an increased risk of T2D (OR = 1.86, 95%CI = 1.20–2.87, *P* = 0.039) and AF (OR = 1.06, 95%CI = 1.01–1.10, *P* = 0.046) after the removal of pleiotropic SNPs using MR-PRESSO method.

### Multivariable MR

**Fig. 3:**
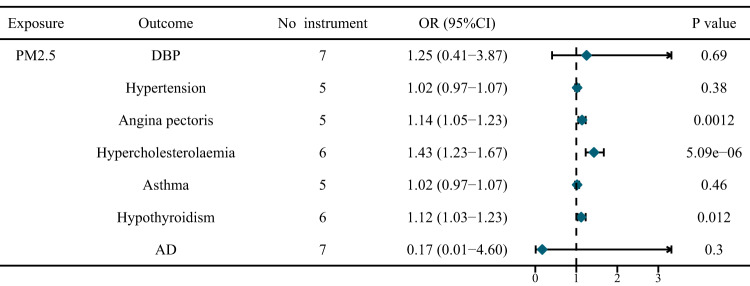
Multivariable MR results adjusting for the effects of other types of air pollution and smoking.

After adjusting for nitrogen dioxide air pollution, nitrogen oxides air pollution, and smoking, genetically predicted PM_2.5_ concentration was associated with an elevated risk of angina pectoris (OR = 1.14, 95%CI = 1.05–1.23, *P* = 0.0012), hypercholesterolaemia (OR = 1.43, 95%CI = 1.23–1.67, *P* = 5.09E-06), and hypothyroidism (OR = 1.12, 95%CI = 1.03–1.23, *P* = 0.012) (Fig. 3, Supplementary Table 4–10). The causal effect sizes of them in multivariable model were all larger than those of univariable model. Interestingly, nitrogen oxides air pollution reduced the risk of hyperemployeolaemia and hypothyroidism in multivariable MR analysis (Supplementary Table 6, 8). Additionally, the multivariate MR showed no direct causal relationship between air pollution and longevity (Supplementary Table 11–12).

### Mediation analysis

PM_2.5_ exposure may affect longevity via potential mediators. Among the 36 potential mediators, we identified the causal relationships between genetically predicted PM_2.5_ concentration and 7 mediators in the first step, and identified the causal effects of DBP, hypertension, hypercholesterolaemia, angina pectoris, hypothyroidism, and AD on longevity in the second step (Table 1, Supplementary Table 13–14). We further evaluated the proportion of indirect effects to the overall effects. The mediation effect of DBP was 0.18 (95%CI = 0.083–0.28, *P* = 0.00029) with a mediated proportion of 31.5% (95% CI, 14.4% to 48.5%) in 90th survival percentile subgroup and 0.21 (95%CI = 0.088 to 0.32, *P* = 0.0006) with a mediated proportion of 28.4% (95% CI, 7.8–18.1%) in 99th survival percentile subgroup (Table 1). The mediation effect of hypertension was −0.41 (95%CI = −0.74 to −0.070, *P* = 0.018) with a mediated proportion of 70.9% (95% CI, 12.2–100%) in 90th percentile subgroup and −0.46 (95%CI = −0.86 to −0.065, *P* = 0.023) with a mediated proportion of 40.9% (95% CI, 5.7–76.0%) in 99th percentile subgroup (Table 1). For every unit increase (1.06 micro-g/m^3^) in PM_2.5_ exposure, the possibility of longevity (top 5%) decreased by 1% via angina pectoris risk (OR = 0.99, 95%CI = 0.97 to 1.00; mediated proportion = 2.5%, 95% CI = 0.4–4.5%; *P* = 0.02) and the possibility of longevity (top 1%) decreased by 2% via angina pectoris risk (OR = 0.98, 95%CI = 0.97–1.00; mediated proportion = 1.5%, 95% CI = 0.2 to 2.9%; *P* = 0.026). The mediation effects of hypercholesterolaemia and AD were significant only in 90th survival percentile subgroup.

**Table 1:** The mediation effect of PM_2.5_ concentration on longevity via potential mediators.

Mediator	Longevity	Total effect β (95% CI)	Direct effect A β (95% CI)	Direct effect B β (95% CI)	Mediation effect β (95% CI)	*P* value	Mediated proportion (%)
DBP	90th	−0.57 (−2.12 to 0.97)	−2.65 (−3.97 to -1.32)	-0.068 (−0.082 to −0.054)	0.18 (0.083 to 0.28)	0.00029	31.48 (14.44 to 48.51)
DBP	99th	−1.13 (−3.55 to 1.28)	−2.65 (−3.97 to −1.32)	−0.077 (−0.099 to −0.056)	0.21 (0.088 to 0.32)	0.00060	28.42 (7.76 to 18.09)
Hypertension	90th	−0.57 (−2.12 to 0.97)	0.081 (0.016 to 0.15)	−5.01 (−6.09 to −3.92)	−0.41(−0.74 to −0.070)	0.018	70.86 (12.23 to 100)
Hypertension	99th	−1.13 (−3.55 to 1.28)	0.081 (0.016 to 0.15)	−5.70 (−7.52 to −3.87)	−0.46 (−0.86 to −0.065)	0.023	40.86 (5.70 to 76.02)
Hypercholesterolaemia	90th	−0.57 (−2.12 to 0.97)	0.072 (0.0070 to 0.14)	−9.61 (−12.95 to −6.27)	−0.69 (−1.36 to −0.023)	0.043	100 (3.92 to 100)
Hypercholesterolaemia	99th	−1.13 (−3.55 to 1.28)	0.072 (0.0070 to 0.14)	−13.13 (−18.87 to −7.38)	−0.94 (−1.89 to 0.0029)	0.051	83.18 (0 to 100)
Angina pectoris	90th	−0.57 (−2.12 to 0.97)	0.045 (0.011 to 0.078)	−0.36 (−0.50 to −0.21)	−0.014 (−0.026 to −0.0022)	0.020	2.45 (0. 39 to 4.50)
Angina pectoris	99th	−1.13 (−3.55 to 1.28)	0.045 (0.011 to 0.078)	−0.39 (−0.58 to −0.20)	−0.017 (−0.033 to −0.0020)	0.026	1.53 (0. 18 to 2.88)
Hypothyroidism	90th	−0.57 (−2.12 to 0.97)	0.092 (0.060 to 0.13)	−3.69 (−7.27 to −0.11)	−0.34 (−0.69 to 0.011)	0.058	59.37 (0 to 100)
Hypothyroidism	99th	−1.13 (−3.55 to 1.28)	0.092 (0.060 to 0.13)	−1.48 (−7.23 to 4.27)	−0.14 (−0.67 to 0.40)	0.62	12.05 (0 to 59.16)
AD	90th	−0.57 (−2.12 to 0.97)	0.48 (0.014 to 0.96)	−0.29 (−0.36 to −0.22)	−0.14 (−0.28 to −0.00016)	0.05	24.65 (0 to 49.27)
AD	99th	−1.13 (−3.55 to 1.28)	0.48 (0.014 to 0.96)	−0.39 (−0.50 to −0.28)	−0.19 (−0.38 to 0.0026)	0.053	16.61 (0 to 33.46)

## Discussion

Long-term exposure to air pollutants has been shown to have a detrimental effect on human life expectancy, potentially leading to premature death^29^. However, little is known about the mediating pathway by which PM_2.5_ affects longevity. In the absence of large-scale RCTs, MR studies that are qualitatively consistent with the results of RCTs can be used for causal inference. To investigate the causal relationship between genetically predicted PM_2.5_ exposure and longevity, we used genetic instrumental variables as proxies. Our primary analyses indicated that, although the association between PM_2.5_ concentration and longevity was not significant, genetically predicted PM_2.5_ increased the risk of hypertension, hypercholesterolaemia, angina pectoris, hypothyroidism and AD, and thus decreased the likelihood of longevity. To account for potential confounders such as other types of air pollution and smoking, we conducted a multivariate MR model and further identified three significant mediators: angina pectoris, hypercholesterolaemia, and hypothyroidism.

Previous observational evidence has demonstrated the gene-environment interaction between longevity genes and air pollution, with certain alleles being more vulnerable to air pollution. For instance, *SIRT1*_391 (rs3758391) allele carriers counteract the detrimental effect of PM_2.5_ exposure and reduce the risk of premature mortality by 26.1%^15^. Similarly, *FOXO3* rs2802292 G allele carriers are protected from the dangers of PM_2.5_ exposure^17^. Since both air pollution and longevity are affected by genetic factors, the association between them can be inferred using genetic variants as proxy. Compared to observational studies, MR method is novel and effective, but needs to meet the three assumptions^27^. Choosing genetic instruments associated with PM_2.5_ concentration is easily achievable, but the other two assumptions need to be tested using statistical methods. Assumption 2 requires that genetic instruments be independent of confounding factors. These PM_2.5_-related SNP instruments may be associated with other types of air pollution and smoking, although the alleles of these SNP established during meiosis and fertilization have avoided the effects of acquired confounding factors. Therefore, we used multivariate MR to adjust for potential confounders (nitrogen dioxide air pollution, nitrogen oxides air pollution, and smoking). For assumption 3, we used MR-Egger to perform a pleiotropy test, and used MR-PRESSO to delete the potential pleiotropic SNP instruments. After adjustment, the results of IVW analysis were basically replicated. Among the non-significant causal estimates in IVW analysis, genetically predicted PM_2.5_ concentration significantly increased the risk of T2D (OR = 1.86, 95%CI = 1.20 to 2.87, *P* = 0.039) and AF (OR = 1.06, 95%CI = 1.01 to 1.10, *P* = 0.046), consistent with previous observational findings.

PM_2.5_ exposure has been shown to be associated with an increased risk of cardiometabolic risk factors and cardio-cerebrovascular diseases^9^. Cross-sectional evidence from the China Health and Retirement Longitudinal Study (CHARLS) among 19,529 participants has demonstrated that an increase in PM_2.5_ concentration is significantly associated with a higher prevalence of hypertension (OR = 1.07, 95% CI = 1.03 to 1.11) and diabetes (OR = 1.15, 95% CI = 1.10 to 1.20), with the impact being relatively stronger in nonsmokers than smokers^30^. Additionally, exposure to both household and outdoor air pollutants has been linked to an increased risk of angina pectoris and myocardial infarction (MI)^31,32^. Recent evidence has also suggested a significant association between PM_2.5_ concentration and dementia^3,33^. A nationwide population-based cohort study conducted by Shi et al. revealed that an interquartile range increase in PM_2.5_ exposure was associated with a 9% increase in AD risk^3^. Among the constituents of PM_2.5_, black carbon (BC) and sulfate (SO_4_^2−^) showed the strongest associations. Interestingly, even improving ambient air quality in late life was associated with a significant reduction in dementia risk, indicating that PM_2.5_ exposure-induced damage to the aging brain may be reversible^33^.

Nevertheless, the exact mechanism by which PM_2.5_ affects chronic disease and longevity in humans remains unclear. It is likely that PM_2.5_ deposits in the lungs, promoting aging through oxidative stress and immune responses^6,34^. Additionally, smaller air pollution particles may enter the bloodstream through different transport routes and mechanisms, leading to toxicity beyond the lung, including potential neurotoxic consequences^35^. Thus, long-term exposure to PM_2.5_ may have a significant impact on the health of a variety of human tissues and organs.

We acknowledge that our study has several limitations. The GWAS for several potential mediators are derived from UK biobank, which have certain sample overlap with the cohort of air pollution. However, there are no other data sources for these phenotypes. Next, genetically predicted PM_10_ and PM_2.5-10_ do not have genome-wide significant genetic variants as instruments, making it difficult to perform MR analysis. Another limitation is that there may be quantitative differences between MR and observational studies or RCTs that should not be interpreted directly as the estimated impact of interventions^36^. Burgess et al. suggest that MR estimates are usually larger than those of observational studies^36^. Additionally, the IVW method usually provides the most effective causal estimates. Deletion of pleiotropic genetic instruments by MR-PRESSO method may result in reduced power or overly precise causal estimates. Therefore, the causal effects of genetically predicted PM_2.5_ on T2D and AF may only be considered suggestive. Finally, power represents a common challenge in the investigation of gene-environment interactions^37,38^. The genetically determined impact of PM_2.5_ on longevity might only constitute a fraction of the total influence. Consequently, it is anticipated that forthcoming research endeavors will address this limitation by augmenting the sample size and enhancing the application of statistical methodologies.

In conclusion, exposure to PM_2.5_ has been linked to an increased risk of hypertension, hypercholesterolaemia, angina pectoris, hypothyroidism and AD, thus having a detrimental effect on longevity. Interventions to reduce environmental PM_2.5_ concentrations are likely to have a significant impact on public health.

## Methods

### MR model

We applied a MR design to investigate the causal effect of PM_2.5_ concentration on longevity and whether potential mediators played a mediating role (Fig. 1). To ensure that the causal estimate is valid, three assumptions must be met: (1) the SNP instruments are significantly associated with exposure, (2) the SNP instruments are not associated with any potential confounder, and (3) the SNP instruments do not affect outcome independently of exposure.

### Data sources

The GWAS summary statistics in this study was publicly available and ethical approval was obtained in all original studies.

### Exposure

GWAS data for PM_2.5_ concentration was obtained from UK Biobank and was as a part of the European Study of Cohorts for Air Pollution Effects (ESCAPE)^39^. A land use regression (LUR) model was used to model for each address between 26 Jan 2010 to 18 January 2011, and air pollution estimates were representative for the year 2010^39,40^. By 2010, the study included 423,796 samples. The mean value of PM_2.5_ concentration was 9.99 micro-g/m^3^ and the standard deviation (SD) was 1.06.

### Outcome

Longevity outcome was derived from a GWAS meta-analysis for age of survival of participants from 20 cohorts of European, East Asian, and African American populations^41^. Cases were participants who lived to an age above the 90th (11,262 cases) or 99th percentile (3,484 cases) based on cohort life tables^41^. Controls were participants who died at or before the age at the 60th percentile. and the 99th survival percentile (25,483 controls)^41^. For instance, the 60th, 90th, and 99th percentile correspond to ages of 83, 94, and 102 years for women in the United States cohort^41^.

### Potential confounders

Other types of air pollution and smoking were identified as potential confounding factors. Data for nitrogen dioxide air pollution, nitrogen oxides air pollution, PM_10_, and PM_2.5-10_ were obtained from UK biobank^39^. GWAS for PM_10_ and PM_2.5-10_ had no genome-wide significant genetic variants. GWAS for cigarettes smoked per day was derived from GWAS & Sequencing Consortium of Alcohol and Nicotine use (GSCAN) containing 337,334 participants^42^.

### Potential mediators

Previous observational studies have reported some potential outcomes of PM_2.5_ exposure, including cardiometabolic risk factors, cardio-cerebrovascular diseases, lung function, autoimmune diseases, and dementia (Table 2)^2–6,27,40,43–46^. These phenotypes were considered as potential mediators. GWAS data for BMI (*N* = 693,529), WHR (*N* = 693,529) after adjusting for BMI, HC (*N* = 142,762) after adjusting for BMI, and WC (*N* = 142,762) after adjusting for BMI were obtained from the genetic investigation of anthropometric traits (GIANT) consortium^47,48^. GWAS for T1D were obtained from the Common Metabolic Diseases Knowledge Portal (CMDKP) and T2D from the Diabetes Meta-Analysis of Trans-Ethnic association studies (DIAMANTE) Consortium^49,50^. GWAS for fasting glucose (FG), fasting insulin (FI) and glycated hemoglobin (HbA1c) were obtained from a meta-analysis, including 281,416 individuals without diabetes (~70% were of European ancestry)^51^. GWAS for LDL-C was obtained from a GWAS meta-analysis of UK Biobank (*n* = 431,167) and the Global Lipids Genetics Consortium (*n* = 188,577)^52^. GWAS for DBP and SBP were obtained from the International Consortium of Blood Pressure (ICBP) (*N* = 757,601). Data for CAD were obtained from a GWAS meta-analysis of nine European cohorts (86,847 cases and 417,789 controls)^53^. Data for HF was derived from a GWAS comprising 47,309 cases and 930,014 controls across 26 studies from the Heart Failure Molecular Epidemiology for Therapeutic Targets (HERMES) Consortium^54^. GWAS for AF (55,114 cases and 482,295 controls) contained more than 50 studies and most of the studies were part of the Atrial Fibrillation Genetics (AFGen) consortium and the Broad AF Study (Broad AF)^55^. Data for stroke was derived from a GWAS meta-analysis of 29 population-based cohorts or biobanks by Mishra et al.^56^. Data for lung function (FVC and FEV1/FVC ratio) were obtained from a meta-analysis of UK biobank and SpiroMeta Consortium (*N* = 321,047)^57^. Data for COPD in never smokers were obtained from UK biobank and were replicated in COPDGene and SpiroMeta Consortium by Kim et al.^58^. GWAS for lung cancer were obtained from a meta-analysis of four GWAS cohorts of lung cancer by Wang et al. from the International Lung Cancer Consortium (ILCCO) (11,348 cases and 15,861 controls)^59^. GWAS for Inflammatory bowel diseases (IBD), including UC and CD, were derived from the International Inflammatory Bowel Disease Genetics Consortium (IIBDGC) (86,640 European individuals)^60^. GWAS data for SLE was obtained from a GWAS meta-analysis including 7,219 cases and 15,991 controls of European ancestry^61^. GWAS for MS was derived from International Multiple Sclerosis Genetics Consortium (IMSGC) including 47,429 cases and 68,374 controls^62^. GWAS for RA were obtained from a GWAS meta-analysis by Ha et al., including 14,361 cases and 43,923 controls^63^. GWAS for AD was derived from a GWAS meta-analysis of GWAS-by-proxy (GWAX) for family history of AD in UK Biobank (53,042 cases and 355,900 controls) with the latest GWAS for diagnosed AD (21,982 cases and 41,944 controls)^64^. GWAS data for ALS was derived from a GWAS analysis from Nicolas et al. containing 20,806 ALS cases and 59,804 controls^65^. GWAS for LBD came from a cohort of 2,981 patients diagnosed with LBD (1,789 autopsy-confirmed LBD cases and 802 clinical LBD cases) and 4,391 controls from 17 European and 27 North American sites/consortia^66^. GWAS data for PD was derived from International Parkinson’s Disease Genomics Consortium (IPDGC), including 33,674 cases and 449,056 controls excluding the 23andMe samples^67^. GWAS for hypercholesterolaemia, hypertension, angina pectoris, IHD, asthma, and hypothyroidism were derived from UK biobank^39^. All the participants of above studies were of European ancestry, and more details were shown in original studies and Supplementary materials. All the participants of above studies were of European ancestry.

**Table 2:** GWAS summary statistics: source and description.

Type	Phenotype	Data source	Sample	Ethnicity
Exposure	PM2.5	UK Biobank/ ESCAPE	423,796	European
Outcome	Longevity	Longevity Genomics research group	11,262/3,484 cases and 25,483 controls	European, East Asian, and African American
Potential confounders	nitrogen dioxide air pollution	UK Biobank/ ESCAPE	456,380	European
	nitrogen oxides air pollution	UK Biobank/ ESCAPE	456,380	European
	PM10	UK Biobank/ ESCAPE	423,796	European
	PM2.5-10	UK Biobank/ ESCAPE	423,796	European
	cigarettes smoked per day	GSCAN	337,334	European
Potential mediators	BMI	GIANT	693,529	European
	WHR	GIANT	693,529	European
	HC	GIANT	142,762	European
	WC	GIANT	142,762	European
	T1D	CMDKP	9,358 cases/15,705 controls	European
	T2D	DIAMANTE	80,154 cases /853,816 controls	European
	FG, FI, HbA1c	GWAS Catalog	281,416	European
	LDL-C	UK Biobank, Global Lipids Genetics Consortium	431,167	European
	Hypercholesterolaemia	UK Biobank	22,622 cases/440,388 controls	European
	DBP	ICBP	757,601	European
	SBP	ICBP	757,601	European
	Hypertension	UK Biobank	2095 cases/460,838 controls	European
	CAD	CMDKP	86,847 cases/417,789 controls	European
	Angina pectoris	UK Biobank	4256 cases/458,754 controls	European
	HF	HERMES	47,309 cases/930,014 controls	European
	AF	CMDKP	55,114 cases/482,295 controls	European
	IHD	UK Biobank	1195 cases/461,815 controls	European
	Stroke	GWAS Catalog	73,652 cases/1,234,808 controls	European
	IS	GWAS Catalog	62,100 cases/1,234,808 controls	European
	FVC	SpiroMeta Consortium, UK Biobank	321,047	European
	FEV1/FVC	SpiroMeta Consortium, UK Biobank	321,047	European
	COPD	UK Biobank, COPDGene Study, SpiroMeta Consortium	21,077 cases/179,689 controls	European
	Lung cancer	ILCCO	11,348 cases/15,861 controls	European
	Asthma	UK Biobank	1877 cases/461,133 controls	European
	Hypothyroidism	UK Biobank	22,687 cases/440,246 controls	European
	SLE	GWAS Catalog	7219 cases/15,991 controls	European
	CD	IIBDGC	17,897 cases/33,977 controls	European
	UC	IIBDGC	13,768 cases/33,977 controls	European
	RA	GWAS Catalog	14,361 cases/43,923 controls	European
	MS	IMSGC	47,429 cases/68,374 controls	European
	ALS	GWAS Catalog	20,806 cases/59,804 controls	European
	PD	IPDGC	33,674 cases/449,056 controls	European
	AD	UK Biobank, IGAP	75,024 cases/397,844 controls	European
	LBD	GWAS Catalog	2981 cases/4,391 controls	European

AD Alzheimer’s disease, AF atrial fibrillation, ALS amyotrophic lateral sclerosis, BMI body mass index, CAD coronary artery disease, CD Crohn’s disease, COPD chronic obstructive pulmonary disease, DBP diastolic blood pressure, FEV1/FVC 1 s forced expiratory volume/FVC, FG fasting glucose, FI fasting insulin, FVC forced vital capacity, HbA1c glycated hemoglobin, HC hip circumference, HF heart failure, IHD ischemic heart disease, IS ischemic stroke, LBD lewy body dementia, MS multiple sclerosis, PD Parkinson’s disease, RA rheumatoid arthritis, SBP systolic blood pressure, SLE Systemic lupus erythematosus, T1D type 1 diabetes, T2D type 2 diabetes, UC Ulcerative colitis, WC waist circumference, WHR waist-hip ratio.

### Selection of instrumental variables

We selected independent genome-wide significant single-nucleotide polymorphisms (SNPs) associated with exposures as genetic instruments (*P* < 5 E-08). The instruments were clumped based on the European 1000 genomes reference panel using PLINK (r^2^ < 0.001). The instruments of palindromic and incompatible alleles were removed when harmonizing exposure and outcome. F statistic < 10 indicated a weak instrument bias in MR analysis.

### Univariable MR

We used IVW as a primary approach to assess the causal effect of genetically predicted PM_2.5_ concentration on longevity, namely combining the Wald ratio estimates of each SNP instrument^27,68–71^. We supplemented IVW method with weighted median estimators, which allowed more powerful instruments to contribute more^27^.

### Sensitivity analysis

If the SNP instruments show horizontal pleiotropy, the MR assumptions may be violated and the MR results may be severely biased^36^. We performed conservative analyses (including fewer variants) to remove the influence of pleiotropy using MR-Egger and MR-PRESSO^27,72^. MR-Egger allows all SNP instruments to have pleiotropic effects, but the pleiotropy effects should be independent of the SNP-exposure association^36^. MR-PRESSO method removes SNP instruments from the analysis whose causal estimates differ substantially from those of other instruments and then continues to perform IVW analysis^36^.

### Multivariable MR

Other types of air pollution and smoking may be confounding factors for the effect of PM_2.5_ on longevity. Multivariable MR allows SNP instruments to be associated with more than one exposure, and estimates the direct causal effect of each exposure in a single MR model^27,36^. We performed multivariate MR analysis to assess the independent causal effect of genetically predicted PM_2.5_ concentration on potential mediators and longevity. The multivariate IVW was used as the primary analysis.

### Mediation analysis

We applied a two-step MR model to calculate the mediation effect of potential mediators. In the first step, we used SNP instruments for PM_2.5_ to estimate the causal effect of PM_2.5_ concentration on potential mediators. In the second step, we used SNP instruments for potential mediating phenotypes to estimate the causal effect of potential mediators on longevity. We assessed the indirect effect of PM_2.5_ concentration on longevity via each mediating factor using product of coefficients method^73–75^. The standard error for the indirect effect was derived by using the delta method^76^.

R packages TwoSampleMR (version 0.5.6) and MRPRESSO (version 1.0) were used for MR analyses. The statistically significant association is defined to be *P* < 0.05/36 = 0.0014 after multiple testing.

### Reporting summary

Further information on research design is available in the Nature Research Reporting Summary linked to this article.

### Supplementary information

SUPPLEMENTAL MATERIAL

Reporting Summary

## Data Availability

GWAS for longevity: https://www.longevitygenomics.org/downloads; GWAS for PM_2.5_: https://gwas.mrcieu.ac.uk/datasets/ukb-b-10817/; GWAS for PM_10_: https://gwas.mrcieu.ac.uk/datasets/ukb-b-18469/; GWAS for PM_2.5-10_: https://gwas.mrcieu.ac.uk/datasets/ukb-b-12963/; GWAS for BMI, WHR, HC, and WC: https://portals.broadinstitute.org/collaboration/giant/index.php/GIANT_consortium_data_files; GWAS for T1D and T2D: https://hugeamp.org/downloads.html; GWAS for FG, FI, HbA1c, LDL-C, hypercholesterolaemia, DBP, SBP, hypertension, IHD, stroke, IS, FVC, FEV1/FVC, COPD, lung cancer, asthma, hypothyroidism, SLE, CD, UC, RA, MS, ALS, PD, AD, and LBD were obtained from IEU OpenGWAS project: https://gwas.mrcieu.ac.uk/; GWAS for angina pectoris, HF, CAD, and AF: https://cd.hugeamp.org/downloads.html GWAS Catalog: https://www.ebi.ac.uk/gwas/home.

## Code availability

The codes for MR and MR-PRESSO are publicly available at https://github.com/MRCIEU/TwoSampleMR and https://github.com/rondolab/MR-PRESSO, respectively.
