# Supplementary material for: Mendelian randomization study supports the causal effects of air pollution on longevity via multiple age-related diseases
Source: NPJ Aging. 2023 Dec 19;9(1):29. doi: 10.1038/s41514-023-00126-0 (PMC10730819; doi:10.1038/s41514-023-00126-0)

Supplementary Information

Supplementary Table 1. Information about instrumental variables.

SNP	A1	A2	EAF	Beta	SE	P value	F statistic
rs114708313	T	A	0.07	0.025	0.0045	4.2E-08	30.86
rs12203592	T	C	0.21	0.022	0.0026	6.2E-17	71.6
rs1372504	A	G	0.37	0.012	0.0022	3.1E-08	29.75
rs1537371	A	C	0.5	0.012	0.0021	8.5E-09	32.65
rs6749467	A	G	0.47	-0.012	0.0022	1.4E-08	29.75
rs72642437	T	C	0.004	0.113	0.0191	3.1E-09	35
rs77205736	T	C	0.27	0.014	0.0024	2.1E-08	34.03
rs77255816	T	C	0.04	0.031	0.0057	4.2E-08	29.58

Supplementary Table 2. Univariate MR analysis of the causal effect of genetically determined PM2.5 concentration on longevity.

**Exposure**	**Outcome**	**Method**	**No. SNPs**	**OR (95%CI)**	**P value**
PM2.5	90th survival percentile	IVW	8	0.56 (0.12-2.63)	0.47
		Weighted median	8	1.06 (0.29-3.83)	0.93
		MR Egger	8	2.09 (0.21-20.59)	0.55
		MR-PRESSO	6	0.37 (0.08-1.66)	0.25
PM2.5	99th survival percentile	IVW	8	0.32 (0.03-3.61)	0.36
		Weighted median	8	1.21 (0.15-9.56)	0.86
		MR Egger	8	2.18 (0.03-161.51)	0.73
		MR-PRESSO	7	0.82 (0.22-3.09)	0.78

Supplementary Table 3. Univariate MR analysis of the causal effect of genetically determined PM2.5 concentration on potential mediators.

Exposure	Outcome	Method	SNP	OR (95%CI)	P value
PM2.5	BMI	IVW	2	1.21 (0.39-3.81)	0.74
	WC	IVW	5	1.31 (0.90-1.90)	0.15
		Weighted median	5	1.51 (1.05-2.19)	0.028
		MR Egger	5	2.17 (0.24-19.50)	0.54
	HC	IVW	5	1.08 (0.77-1.52)	0.65
		Weighted median	5	1.02 (0.69-1.51)	0.93
		MR Egger	5	1.46 (0.17-12.33)	0.75
	WHR	IVW	5	1.25 (0.87-1.80)	0.22
		Weighted median	5	1.36 (0.94-1.97)	0.11
		MR Egger	5	2.69 (0.33-21.69)	0.42
	T1D	IVW	8	1.64 (0.48-5.67)	0.43
		Weighted median	8	2.31 (0.45-11.93)	0.32
		MR Egger	8	5.00 (0.34-74.59)	0.29
	T2D	IVW	8	1.80 (0.89-3.64)	0.1
		Weighted median	8	1.99 (1.22-3.25)	0.0058
		MR Egger	8	1.46 (0.35-6.11)	0.62
		MR-PRESSO	6	1.86 (1.20-2.87)	0.039
	FI	IVW	8	1.03 (0.92-1.16)	0.58
		Weighted median	8	1.00 (0.91-1.11)	0.94
		MR Egger	8	0.99 (0.83-1.18)	0.92
	FG	IVW	7	0.99 (0.91-1.07)	0.73
		Weighted median	7	0.98 (0.89-1.07)	0.6
		MR Egger	7	0.99 (0.88-1.10)	0.81
	HbA1c	IVW	8	1.01 (0.91-1.11)	0.92
		Weighted median	8	1.04 (0.96-1.13)	0.31
		MR Egger	8	1.07 (0.92-1.24)	0.42

LDL-C	IVW	8	0.99 (0.76-1.29)	0.96
	Weighted median	8	0.95 (0.81-1.11)	0.5
	MR Egger	8	1.13 (0.57-2.24)	0.74
	MR-PRESSO	5	1.03 (0.90-1.17)	0.7
Hypercholesterolaemia	IVW	7	1.07 (1.01-1.15)	0.03
	Weighted median	7	1.05 (1.02-1.09)	0.0041
	MR Egger	7	0.99 (0.80-1.24)	0.96
	MR-PRESSO	6	1.04 (1.03-1.06)	0.0047
SBP	IVW	7	0.10 (0.00-7.67)	0.3
	Weighted median	7	0.10 (0.01-1.42)	0.089
	MR Egger	7	0.00 (0.00-11025.05)	0.47
	MR-PRESSO	5	0.03 (0.00-0.64)	0.088
DBP	IVW	7	0.07 (0.02-0.27)	9.24E-05
	Weighted median	7	0.12 (0.03-0.50)	0.004
	MR Egger	7	0.03 (0.00-3.26)	0.2
Hypertension	IVW	7	1.08 (1.02-1.16)	0.014
	Weighted median	7	1.08 (1.03-1.14)	0.0026
	MR Egger	7	1.00 (0.80-1.24)	0.99
CAD	IVW	8	1.99 (0.16-24.74)	0.59
	Weighted median	8	0.94 (0.71-1.23)	0.64
	MR Egger	8	0.38 (0.01-16.30)	0.63
	MR-PRESSO	3	1.56 (0.85-2.86)	0.29
Angina pectoris	IVW	5	1.05 (1.01-1.08)	0.0084
	Weighted median	5	1.03 (1.01-1.05)	0.000777
	MR Egger	5	1.02 (0.87-1.20)	0.81
	MR-PRESSO	4	1.03 (1.02-1.04)	0.0093
HF	IVW	7	2.42 (0.67-8.75)	0.18
	Weighted median	7	1.15 (0.64-2.05)	0.64
	MR Egger	7	0.86 (0.09-8.33)	0.9

AF	IVW	8	1.30 (0.72-2.35)	0.39
	Weighted median	8	1.04 (0.65-1.64)	0.88
	MR Egger	8	0.71 (0.27-1.90)	0.52
	MR-PRESSO	6	1.06 (1.01-1.10)	0.046
IHD	IVW	4	1.01 (0.99-1.04)	0.37
	Weighted median	4	1.01 (1.00-1.03)	0.05
	MR Egger	4	1.13 (0.54-2.37)	0.78
	MR-PRESSO	2	1.01 (1.01-1.02)	0.11
Stroke	IVW	8	1.74 (0.62-4.93)	0.3
	Weighted median	8	1.20 (0.61-2.36)	0.6
	MR Egger	8	0.45 (0.08-2.53)	0.4
IS	IVW	8	1.16 (0.62-2.19)	0.64
	Weighted median	8	0.95 (0.69-1.30)	0.74
	MR Egger	8	0.76 (0.36-1.61)	0.5
Lung function (FVC)	IVW	8	0.94 (0.79-1.11)	0.45
	Weighted median	8	1.02 (0.85-1.22)	0.86
	MR Egger	8	0.92 (0.61-1.37)	0.69
Lung function (FEV1/FVC)	IVW	8	0.91 (0.74-1.11)	0.36
	Weighted median	8	0.92 (0.76-1.12)	0.39
	MR Egger	8	0.62 (0.44-0.86)	0.03
COPD	IVW	6	3.20 (0.63-16.19)	0.16
	Weighted median	6	5.95 (1.57-22.53)	0.0087
	MR Egger	6	6.15 (0.02-2340.21)	0.58
Lung cancer	IVW	7	0.43 (0.15-1.26)	0.12
	Weighted median	7	0.67 (0.25-1.83)	0.44
	MR Egger	7	0.80 (0.17-3.84)	0.8
Asthma	IVW	5	1.01 (1.00-1.02)	0.011
	Weighted median	5	1.01 (1.00-1.02)	0.15

	MR Egger	5	1.06 (1.02-1.10)	0.063
SLE	IVW	7	0.45 (0.05-4.16)	0.48
	Weighted median	7	2.40 (0.32-18.13)	0.4
	MR Egger	7	3.65 (0.21-64.47)	0.42
RA	IVW	8	0.78 (0.35-1.73)	0.54
	Weighted median	8	0.93 (0.67-1.30)	0.68
	MR Egger	8	1.03 (0.36-2.94)	0.96
CD	Wald ratio	1	1.91 (0.27-13.71)	0.52
UC	IVW	7	0.59 (0.26-1.33)	0.21
	Weighted median	7	0.82 (0.29-2.34)	0.71
	MR Egger	7	0.47 (0.11-1.98)	0.35
MS	IVW	6	2.21 (0.58-8.36)	0.24
	Weighted median	6	1.03 (0.19-5.66)	0.98
	MR Egger	6	0.26 (0.00-17.79)	0.57
Hypothyroidism	IVW	7	1.10 (1.06-1.13)	2.93E-08
	Weighted median	7	1.10 (1.06-1.14)	1.09E-06
	MR Egger	7	1.12 (1.00-1.25)	0.12
AD	IVW	8	1.62 (1.01-2.60)	0.044
	Weighted median	8	1.49 (0.82-2.69)	0.19
	MR Egger	8	1.83 (0.74-4.49)	0.24
PD	IVW	8	0.90 (0.23-3.45)	0.88
	Weighted median	8	1.51 (0.39-5.95)	0.55
	MR Egger	8	4.92 (0.21-114.42)	0.35
ALS	IVW	8	1.23 (0.89-1.72)	0.22
	Weighted median	8	1.89 (1.20-2.96)	0.0056
	MR Egger	8	1.78 (0.96-3.31)	0.077
LBD	IVW	6	1.51 (0.15-15.09)	0.73
	Weighted median	6	1.08 (0.05-22.41)	0.96
	MR Egger	6	0.00 (0.00-8.70)	0.23

Supplementary Table 4. Causal effects of genetically determined PM2.5 concentration, cigarettes smoked per day, nitrogen oxides air pollution and nitrogen dioxide air pollution on DBP using multivariable MR analysis.

**Exposure**	**Outcome**	**No. SNPs**	**beta**	**se**	**P value**
PM2.5	DBP	7	0.226	0.575	0.694
Cigarettes smoked per day		22	0.001	0.020	0.980
Nitrogen oxides air pollution		6	0.589	1.090	0.589
Nitrogen dioxide air pollution		7	-1.033	0.871	0.236

Supplementary Table 5. Causal effects of genetically determined PM2.5 concentration, cigarettes smoked per day, nitrogen oxides air pollution and nitrogen dioxide air pollution on angina pectoris using multivariable MR analysis.

Exposure	Outcome	No. SNPs	beta	se	P value
PM2.5	Angina pectoris	5	0.127	0.039	0.0012
Cigarettes smoked per day		20	-0.001	0.001	0.50
Nitrogen oxides air pollution		6	-0.080	0.075	0.29
Nitrogen dioxide air pollution		5	-0.028	0.061	0.65

Supplementary Table 6. Causal effects of genetically determined PM2.5 concentration, cigarettes smoked per day, nitrogen oxides air pollution and nitrogen dioxide air pollution on hypercholesterolaemia using multivariable MR analysis.

**Exposure**	**Outcome**	**No. SNPs**	**beta**	**se**	**P value**
PM2.5	Hypercholesterolaemia	6	0.357	0.078	5.09E-06
Cigarettes smoked per day		22	0.006	0.003	0.018
Nitrogen oxides air pollution		6	-0.364	0.145	0.012
Nitrogen dioxide air pollution		7	0.024	0.115	0.83

Supplementary Table 7. Causal effects of genetically determined PM2.5 concentration, cigarettes smoked per day, nitrogen oxides air pollution and nitrogen dioxide air pollution on hypertension using multivariable MR analysis.

**Exposure**	**Outcome**	**No. SNPs**	**beta**	**se**	**P value**
PM2.5	hypertension	5	0.0211	0.0242	0.38
Cigarettes smoked per day		17	-0.000511	0.000778	0.51
Nitrogen oxides air pollution		5	-0.0359	0.0435	0.41
Nitrogen dioxide air pollution		3	0.0161	0.0377	0.67

Supplementary Table 8. Causal effects of genetically determined PM2.5 concentration, cigarettes smoked per day, nitrogen oxides air pollution and nitrogen dioxide air pollution on hypothyroidism using multivariable MR analysis.

**Exposure**	**Outcome**	**No. SNPs**	**beta**	**se**	**P value**
PM2.5	hypothyroidism	6	0.117	0.046	0.012
Cigarettes smoked per day		21	0.00261	0.002	0.104
Nitrogen oxides air pollution		6	-0.223	0.090	0.013
Nitrogen dioxide air pollution		6	0.117	0.073	0.108

Supplementary Table 9. Causal effects of genetically determined PM2.5 concentration, cigarettes smoked per day, nitrogen oxides air pollution and nitrogen dioxide air pollution on AD using multivariable MR analysis.

**Exposure**	**Outcome**	**No. SNPs**	**beta**	**se**	**P value**
PM2.5	AD	7	-1.748	1.671	0.30
Cigarettes smoked per day		22	-0.036	0.060	0.55
Nitrogen oxides air pollution		6	-0.296	3.269	0.93
Nitrogen dioxide air pollution		7	2.546	2.667	0.34

Supplementary Table 10. Causal effects of genetically determined PM2.5 concentration, cigarettes smoked per day, nitrogen oxides air pollution and nitrogen dioxide air pollution on asthma using multivariable MR analysis.

**Exposure**	**Outcome**	**No. SNPs**	**beta**	**se**	**P value**
PM2.5	asthma	5	0.0190	0.0260	0.46
Cigarettes smoked per day		17	-0.0006	0.0008	0.43
Nitrogen oxides air pollution		4	0.0192	0.0486	0.69
Nitrogen dioxide air pollution		3	-0.0267	0.0398	0.50

Supplementary Table 11. Causal effects of genetically determined PM2.5 concentration, cigarettes smoked per day, nitrogen oxides air pollution and nitrogen dioxide air pollution on longevity (90th percentile) using multivariable MR analysis.

**Exposure**	**Outcome**	**No. SNPs**	**beta**	**se**	**P value**
PM2.5	longevity	6	-0.854	3.017	0.78
Cigarettes smoked per day		19	-0.127	0.114	0.27
Nitrogen oxides air pollution		6	3.732	6.119	0.54
Nitrogen dioxide air pollution		7	-3.083	5.403	0.57

Supplementary Table 12. Causal effects of genetically determined PM2.5 concentration, cigarettes smoked per day, nitrogen oxides air pollution and nitrogen dioxide air pollution on longevity (99th percentile) using multivariable MR analysis.

**Exposure**	**Outcome**	**No. SNPs**	**beta**	**se**	**P value**
PM2.5	longevity	6	-5.7137	4.695	0.22
Cigarettes smoked per day		19	-0.0001	0.170	0.99
Nitrogen oxides air pollution		6	0.1423	9.235	0.99
Nitrogen dioxide air pollution		7	5.1526	8.118	0.53

Supplementary Table 13. MR analysis of the causal effect of genetically determined asthma on longevity (90th percentile).

Exposure	Outcome	Method	No. SNPs	OR (95%CI)	P value
asthma	90th percentile	IVW	95	1.55 (0.69-3.49)	0.29
		Weighted median	95	1.48 (0.50-4.33)	0.48
		MR Egger	95	0.47 (0.06-3.68)	0.47

Supplementary Table 14. MR analysis of the causal effect of genetically determined asthma on longevity (99th percentile).

Exposure	Outcome	Method	No. SNPs	OR (95%CI)	P value
asthma	99th percentile	IVW	91	0.71 (0.22-2.34)	0.58
		Weighted median	91	1.63 (0.29-9.10)	0.58
		MR Egger	91	1.02 (0.05-21.09)	0.99

Supplementary Figure 1. Scatter plot about the causal effect of PM2.5 concentration on longevity. The x-axis shows the SNP effect and standard error on each of PM2.5 concentration SNPs. The y-axis shows the SNP effect and standard error on longevity.

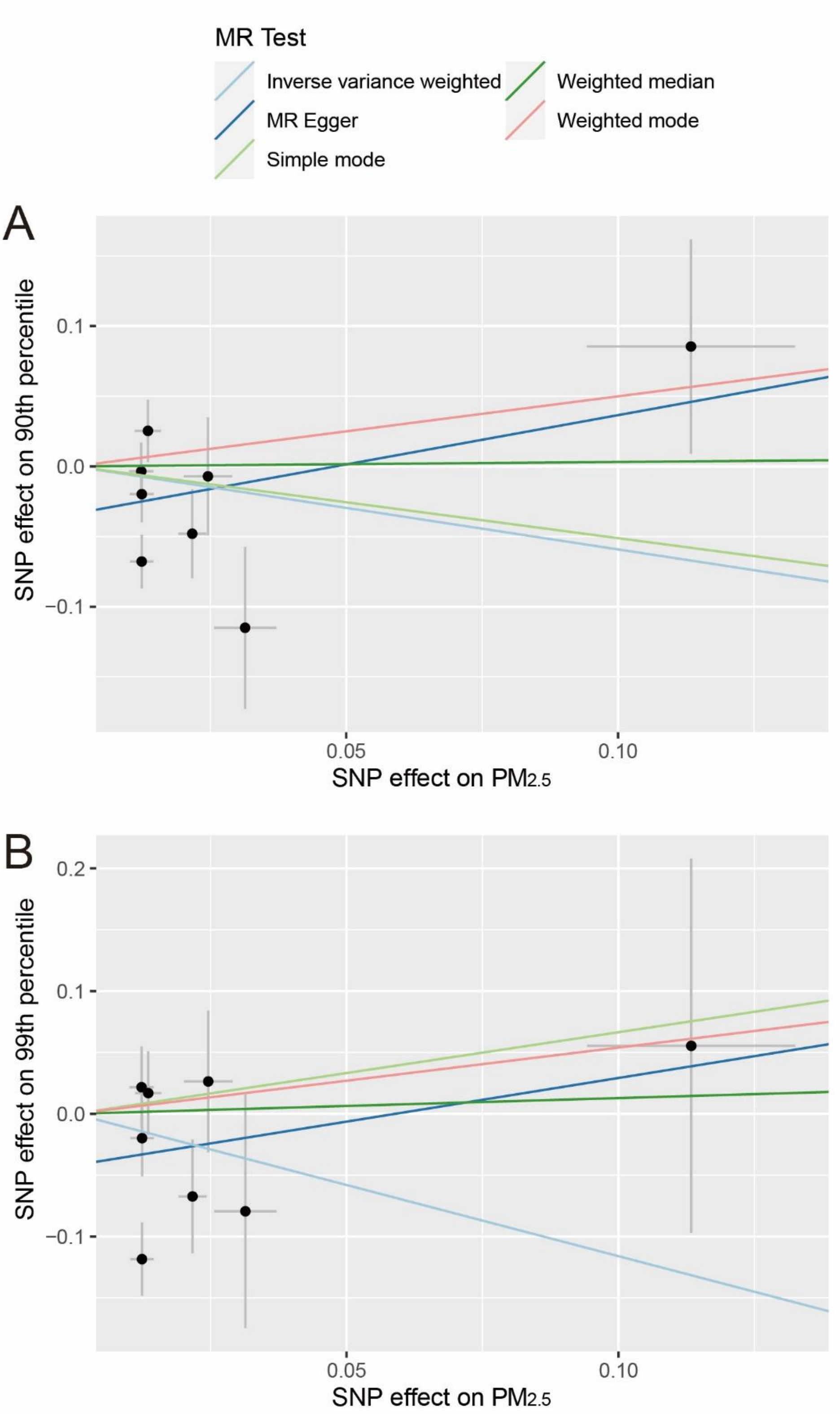

Supplement: Supplementary file 1 — SUPPLEMENTAL MATERIAL [file 41514_2023_126_MOESM1_ESM.pdf]
